# Liver function following hepatitis C virus eradication by direct acting antivirals in patients with liver cirrhosis: data from the PITER cohort

**DOI:** 10.1186/s12879-021-06053-3

**Published:** 2021-05-04

**Authors:** Maria Giovanna Quaranta, Luigina Ferrigno, Xhimi Tata, Franca D’Angelo, Carmine Coppola, Alessia Ciancio, Serena Rita Bruno, Martina Loi, Alessia Giorgini, Marzia Margotti, Valentina Cossiga, Giuseppina Brancaccio, Marcello Dallio, Martina De Siena, Marco Cannizzaro, Luisa Cavalletto, Marco Massari, Maria Mazzitelli, Pasqualina De Leo, Diletta Laccabue, Leonardo Baiocchi, Loreta A. Kondili

**Affiliations:** 1 Center for Global Health, Istituto Superiore di Sanità, Viale Regina Elena 299, 00161 Rome, Italy; 2 University of Tor Vergata, Nostra Signora del Buon Consiglio di Tirana, Tirana, Albania; 3 Department of Hepatology, Gragnano Hospital, Naples, Italy; 4 Gastroenterology Unit, University of Turin, Turin, Italy; 5 Infectious Diseases, Ospedali Riuniti, Foggia, Italy; 6 Liver Unit, University Hospital, Monserrato, Cagliari, Italy; 7 Department of Health Sciences, San Paolo Hospital, University of Milan, Milan, Italy; 8 Department of Internal Medicine, University Hospital of Modena, Modena, Italy; 9 Department of Clinical Medicine and Surgery, Federico II University, Naples, Italy; 10 Department of Infectious Disease, University of Padua, Padua, Italy; 11 Department of Precision Medicine, University of Campania Luigi Vanvitelli, Naples, Italy; 12 Department of Internal Medicine and Gastroenterology, Fondazione Policlinico A. Gemelli IRCCS, Università Cattolica del Sacro Cuore, Rome, Italy; 13 Internal Medicine, Villa Sofia-Cervello Hospital, Palermo, Italy; 14 Department of Medicine, University Hospital of Padua, Padua, Italy; 15 Infectious Diseases, Azienda Unità Sanitaria Locale – IRCCS di Reggio Emilia, Reggio Emilia, Italy; 16 Department of Infectious Disease, University Hospital Mater Domini, Catanzaro, Italy; 17 Department of Infectious Disease, San Paolo Hospital, Savona, Italy; 18 Unit of Infectious Diseases and Hepatology, Azienda Ospedaliero-Universitaria di Parma, Parma, Italy; 19 Department of Medical Sciences, University of Tor Vergata, Rome, Italy

**Keywords:** Hepatitis C virus, Human immunodeficiency virus, Real-life cohort, Direct-acting antivirals, Advanced liver disease, Decompensated cirrhosis

## Abstract

**Background:**

The development of direct-acting antivirals (DAA) for HCV has revolutionized the treatment of HCV, including its treatment in patients with HIV coinfection. The aim of this study was to compare the changes in liver function between coinfected and monoinfected patients with cirrhosis who achieved HCV eradication by DAA.

**Methods:**

Patients with pre-treatment diagnosis of HCV liver cirrhosis, consecutively enrolled in the multicenter PITER cohort, who achieved a sustained virological response 12 weeks after treatment cessation (SVR12) were analysed. Changes in Child-Pugh (C-P) class and the occurrence of a decompensating event was prospectively evaluated after the end of DAA treatment. Cox regression analysis was used to evaluate factors independently associated with changes in liver function following viral eradication.

**Results:**

We evaluated 1350 patients, of whom 1242 HCV monoinfected (median follow-up 24.7, range 6.8–47.5 months after viral eradication) and 108 (8%) HCV/HIV coinfected (median follow-up 27.1, range 6.0–44.6).

After adjusting for age, sex, HCV-genotype, HBsAg positivity and alcohol use, HIV was independently associated with a more advanced liver disease before treatment (C-P class B/C vs A) (OR: 3.73, 95% CI:2.00–6.98). Following HCV eradication, C-P class improved in 17/20 (85%) coinfected patients (from B to A and from C to B) and in 53/82 (64.6%) monoinfected patients (from B to A) (*p* = 0.08). C-P class worsened in 3/56 coinfected (5.3%) (from A to B) and in 84/1024 (8.2%) monoinfected patients (*p* = 0.45) (from A to B or C and from B to C).

Baseline factors independently associated with C-P class worsening were male sex (HR = 2.00; 95% CI = 1.18–3.36), platelet count < 100,000/μl (HR = 1.75; 95% CI 1.08–2.85) and increased INR (HR = 2.41; 95% CI 1.51–3.84). Following viral eradication, in 7 of 15 coinfected (46.6%) and in 61 of 133 (45.8%) monoinfected patients with previous history of decompensation, a new decompensating event occurred. A first decompensating event was recorded in 4 of 93 (4.3%) coinfected and in 53 of 1109 (4.8%) monoinfected patients (*p* = 0.83).

**Conclusions:**

Improvement of liver function was observed following HCV eradication in the majority of patients with cirrhosis; however viral eradication did not always mean cure of liver disease in both monoinfected and coinfected patients with advanced liver disease.

**Supplementary Information:**

The online version contains supplementary material available at 10.1186/s12879-021-06053-3.

## Background

Hepatitis C Virus (HCV) eradication by direct-acting antivirals (DAA) is linked to improved outcomes at all stages of liver disease [1]. Available data suggest that the maximum efficacy and benefits are obtained by treating patients before they reach the stage of advanced fibrosis or cirrhosis [2, 3]. Sustained virologic response (SVR) reduces the risk of liver decompensation and of hepatocellular carcinoma (HCC) and improves survival [1, 4, 5]. However, a “point of non-return” in terms of deterioration of liver function, has been observed in part of patients regardless of viral eradication, potentially due to the pre-treatment severe liver fibrosis and/or presence of other cofactors of liver disease progression [3, 4, 6]. As a cofactor, Human Immunodeficiency Virus (HIV) coinfection negatively affect the natural course of chronic HCV infection. Patients have a faster progression of liver fibrosis, an earlier transition to cirrhosis, a higher risk of hepatic decompensation and occurrence of HCC compared with HCV-monoinfected patients as well as a potential toxicity of antiretroviral therapy in the liver [7–12].

The development of DAA against HCV has revolutionized the treatment of hepatitis C, including its treatment in patients with HIV coinfection, which have resulted with similar SVR rates as those of HCV monoinfected patients, shorter and simpler regimens with minimal treatment-related side effects compared with previous Interferon (IFN)-based therapies [13–17]. However, for HIV/HCV-coinfected patients with cirrhosis, the potential benefits of viral eradication could be counterbalanced by a poorer recovery of liver function. A critical issue for both monoinfected and coinfected patients that eradicate HCV infection in the cirrhosis stage of liver disease is the occurrence or deterioration of liver-related clinical events.

The aim of this study was to assess the liver disease outcomes after viral eradication in terms of changes in Child-Pugh (C-P) class and the occurrence of a decompensating event, in a real-life cohort of patients with advanced liver disease due to HCV infection with or without HIV coinfection.

## Methods

### Patients

Patients were recruited from the Italian Platform for the study of viral hepatitis therapy (PITER) cohort between April 2015 and June 2019. PITER is a prospective multicentric cohort, considered representative of patients with chronic HCV infection in care in Italy. Patients have been consecutively enrolled in certain periods of time yearly and were not receiving HCV treatment at the time of inclusion in the cohort [18]. In this study, HCV/HIV coinfected patients and HCV monoinfected patients with known HIV negative status, with pre-treatment diagnosis of liver cirrhosis who had achieved a SVR 12 weeks after DAA treatment cessation (SVR12) were included. Patients with a history of liver transplantation prior to treatment were excluded. Patients’ data prior to the treatment start were considered as baseline. Patients’ data during the follow-up after the end of treatment were prospectively evaluated. Viral eradication was defined as undetectable HCV-RNA level, as assessed by highly sensitive molecular methods (lower limit of detection ranging from ≤12 to ≤15 IU/ml), at the end of treatment and at the 12-week post-treatment evaluation.

Fibrosis stage was defined based on liver transient elastography data, which were considered as validated if each patient had at least 10 valid stiffness measurements, with a success rate of at least 80%, an interquartile range of less than 30% of the median stiffness score, and a body mass index (BMI) of < 30 kg/m^2^ [19]. Liver cirrhosis was defined when the stiffness score was equal to or higher than 12.5 kPa or according to biochemical and instrumental data of portal hypertension [20].

### Outcome variables

Clinical outcomes evaluated included the occurrence of a decompensating event (ascites and/or gastrointestinal bleeding due to portal hypertension and/or hepatic encephalopathy) and changes in the severity of liver disease in terms of C-P class worsening or improvement whatever occurred first during the follow-up after the end of treatment.

### Statistical analysis

Patient’s main baseline characteristics were reported as median and interquartile range (IQR) or as proportions [number (N) and percentage (%)] for continuous and categorical variables, respectively. The Mann-Whitney U test was used for continuous variables to assess differences between distribution, and the Chi-squared test was used for comparisons of proportions. A *p*-value of < 0.05 was considered statistically significant.

A multiple logistic regression analysis was performed using C-P class prior to antiviral treatment as the dependent variable and the following variables at baseline as covariates: age, sex, HCV genotype, HBsAg positivity, alcohol use and HIV coinfection.

Variables independently associated with worsening of liver function, as determined by changes in C-P class after the end of treatment, were evaluated by Cox proportional hazard models. All analyses were performed using the STATA/SE 15.1 statistical package (StataCorp LP, College Station, TX, USA).

## Results

### Baseline characteristics of patients

A total of 108 HIV/HCV coinfected (81.5% males) and 1242 HCV monoinfected patients (58.1% males) were considered in the present analyses. Baseline characteristics of these patients are summarised in Table 1.

**Table 1:** - Baseline characteristics of cirrhotic patients

		HCV/HIV co-infected (***N*** = 108*)		HCV mono-infected (***N*** = 1242*)		
**Quantitative variables**		Median	IQR	Median	IQR	p**
Age (years)		52.5	50–55	64.0	54–72	**< 0.001**
ALT (IU/L)		63.0	40.0–91.0	74.0	47.0–115.0	**0.01**
AST (IU/L)		60.0	44.0–95.0	70.0	47.0–105.0	0.14
Platelets/μL		105,000	74,000–154,000	119,000	86,350–159,000	0.06
Albumin (g/dL)		3.9	3.5–4.2	3.9	3.6–4.2	0.48
Bilirubin (mg/dL)		0.8	0.6–1.3	0.9	0.6–1.1	0.54
INR		1.1	1.0–1.2	1.1	1.0–1.2	0.10
**Categorical variables**		N.	%	N.	%	p***
Sex	Male	88	81.5	722	58.1	**< 0.001**
	Female	20	18.5	520	41.9	
BMI	Underweight	5	4.6	14	1.1	**< 0.001**
	Normal	70	64.8	514	41.4	
	Overweight	25	23.2	550	44.3	
	Obese	8	7.4	163	13.1	
Alcohol use	Never	50	52.1	803	66.0	**< 0.001**
	Current	26	27.1	116	9.5	
	Past	20	20.8	297	24.4	
HCV- genotype	1 (Non subtyped)	5	4.6	36	2.9	**< 0.001**
	1a	33	30.6	170	13.7	
	1b	15	13.9	665	53.5	
	2	4	3.7	168	13.5	
	3	31	28.7	120	9.7	
	4	20	18.5	83	6.7	
	5	0	0.0	0	0.0	
Diabetes	Yes	16	14.8	259	20.9	0.14
	No	92	85.2	983	79.2	
HBV Infection	Anti-HBc+/HBsAg+	4	3.7	15	1.2	**< 0.001**
	Anti-HBc+/HBsAg-	47	43.5	262	21.1	
	No	57	52.8	965	77.7	
Previous Interferon	Yes	30	27.8	415	33.4	0.23
	No	78	72.2	827	66.6	
HCC	Yes	1	0.9	78	6.3	**0.02**
	No	107	99.1	1164	93.7	
Esophageal varices	Yes	18	16.7	270	21.7	0.22
	No	90	83.3	972	78.3	
Ascites	Yes	10	9.3	81	6.5	0.28
	No	98	90.7	1161	93.5	
Previous decompensation	Yes	15	13.9	133	10.7	0.31
	No	93	86.1	1109	89.3	
Child-Pugh Score	A-5	39	52.7	762	69.5	**< 0.001**
	A-6	14	18.9	242	22.1	
	B-7	12	16.2	58	5.3	
	B-8	8	10.8	28	2.6	
	B-9	0	0.0	6	0.6	
	C-10	1	1.4	0	0.0	

*For some variables inconsistencies are due to missing values

**p value Mann–Whitney rank-sum test

***p value Chi-square test

Coinfected patients were significantly younger (median age of 52.5 vs 64 years, *p* < 0.001) and compared with monoinfected patients had significantly lower BMI (*p* < 0.001). Current alcohol use is more frequently reported in coinfected compared with monoinfected patients (*p* < 0.001).

A significantly different distribution in HCV genotypes in monoinfected compared with coinfected patients was observed. About half of the monoinfected patients (*n* = 665, 53.5%) were infected by HCV genotype 1b, whereas genotype 1a and 3 were prevalent in coinfected patients (*n* = 33, 30.6% and *n* = 31, 28.7%, respectively) (*p* < 0.001).

Higher prevalence of anti-HBc+/HBsAg- (43.5% vs 21.1%) and anti-HBc+/HBsAg+ (3.7% vs 1.2%, *p* < 0.001) was detected in coinfected compared with monoinfected patients (*p* < 0.001).

A significant difference in the prevalence of HCC between coinfected and monoinfected patients was observed (0.9% vs 6.3%, respectively; *p* = 0.02).

Before starting therapy, 15 (13.9%) co-infected and 133 (10.7%) monoinfected patients had reported a previous liver decompensating event (*p* = 0.31).

Coinfected patients had a more severe liver disease in terms of C-P class distribution (A5: 52.7% vs 69.5%; A6: 18.9% vs 22.1%; B7: 16.2% vs 5.3%; B8: 10.8% vs 2.6%), compared with monoinfected patients (*p* < 0.001).

After adjusting for potential factors of liver disease severity before treatment, such as age, sex, HCV genotype, HBsAg positivity, alcohol use and HIV coinfection, HIV was the only factor independently associated [Odds Ratios (OR): 3.73, 95% Confidence Interval (CI): 2.00–6.98] with C-P class B/C vs A (Table 2).

**Table 2:** - Baseline factors associated with Child-Pugh class (A vs B/C). Multivariate analysis

Baseline factors	Adjusted O.R.	95% CI
Age (increasing years)	1.00	0.98–1.02
Sex (ref. female)	1.07	0.69–1.67
Current/past alcohol use (ref. never)	0.87	0.56–1.37
HCV-genotype (3 vs others)	1.48	0.80–2.76
HBsAg+	2.27	0.57–8.99
HIV+	**3.73**	**2.00–6.98**

### Prospective evaluation on changes in liver function after viral eradication

**Fig. 1:**
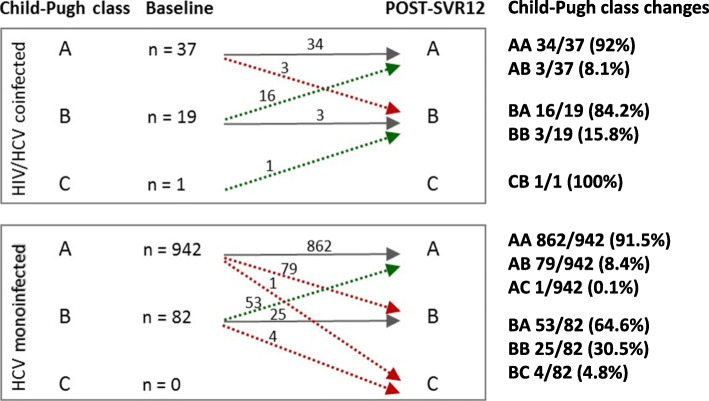
Changes in the severity of liver disease in terms of C-P class improvement or worsening in monoinfected and coinfected patients

Coinfected and monoinfected patients were evaluated during a median follow-up of 27.1 (range 6.0–44.6) and 24.7 (range 6.8–47.5) months after viral eradication, respectively. Changes in the severity of liver disease in terms of C-P class worsening or improvement are shown in Fig. 1.

During the follow-up C-P class improved in 17/20 (85%) coinfected patients (of whom 16 from C-P class B to A and 1 from C-P class C to B) and in 53/82 (64.6%) monoinfected patients (from C-P class B to A) (*p* = 0.08). C-P class worsened in 3/56 (5.3%) coinfected patients (from C-P class A to B) and in 84/1024 (8.2%) HCV-monoinfected patients (of whom 79 from C-P class A to B, 4 from C-P class B to C and 1 from C-P class A to C) (*p* = 0.45).

No difference in the occurrence of a decompensating event following viral eradication was observed between coinfected (*n* = 11, 10.2%) and monoinfected (*n* = 114, 9.2%) (*p* = 0.73) patients. Of patients with decompensated cirrhosis prior to therapy in 7 of 15 (46.6%) coinfected and in 61 of 133 (45.8%) monoinfected patients a new decompensating event occurred following viral eradication. A total of 4 of 93 (4.3%) coinfected and 53 of 1109 (4.8%) (*p* = 0.83) monoinfected patients had their first decompensating event. Decompensating events recorded after viral eradication in both patients with or without a previous history of decompensation were mainly represented by the presence of ascites, however encephalopathy and variceal bleeding were also present (Table 3).

**Table 3:** Occurrence of decompensating event following viral eradication

	HCV/HIV co-infected (***N*** = 11)		HCV mono-infected (***N*** = 114)	
**Patients with a pre-treatment history of decompensated cirrhosis**	**N.**	**%**	**N.**	**%**
Ascites	6	85.7	32	52.5
Ascites + hepatic encephalopathy	0	0.0	8	13.1
Ascites + gastrointestinal bleeding	0	0.0	2	3.3
Ascites + gastrointestinal bleeding + hepatic encephalopathy	0	0.0	2	3.3
Hepatic encephalopathy	0	0.0	9	14.8
Gastrointestinal bleeding	1	14.3	8	13.1
**TOTAL**	**7**	**100.0**	**61**	**100.0**
**Patients with incident decompensating event**	**N.**	**%**	**N.**	**%**
Ascites	3	75.0	32	67.9
Ascites + hepatic encephalopathy	0	0.0	6	3.8
Hepatic encephalopathy	1	25.0	6	11.3
Gastrointestinal bleeding	0	0.0	9	17.0
**TOTAL**	**4**	**100.0**	**53**	**100.0**

### Baseline predictors of Child-Pugh class worsening after viral eradication

Male sex [Hazard ratio (HR) = 1.77; 95% CI: 1.12–2.81], platelet count lower than 100,000/μL (HR = 2.01; 95% CI: 1.31–3.08), increased international normalized ratio (INR) (HR = 2.15; 95% CI: 1.45–3.19), presence of esophageal varices (HR = 1.85; 95% CI: 1.20–2.85), history of HCC (HR = 2.32; 95% CI: 1.20–4.49) and history of decompensation (HR = 1.97; 95% CI: 1.17–3.31) were significantly associated with C-P class worsening at univariate analysis. At multivariate analysis, male sex (HR = 2.00; 95% CI: 1.18–3.36), platelet count lower than 100,000/μL (HR = 1.75; 95% CI: 1.08–2.85) and increased INR (HR = 2.41; 95% CI: 1.51–3.84) resulted independently associated with C-P class worsening. HIV coinfection was not associated with the C-P class worsening both at univariate and at multivariate analysis (Table 4).

**Table 4:** - Baseline factors associated with Child-Pugh class worsening following viral eradication**.** Univariate and multivariate analysis

Baseline factors	Crude HR	95% CI	Adjusted HR	95% CI
HIV infection	0.68	0.21–2.15	0.51	0.15–1.73
Age (increasing years)	1.00	0.98–1.02	1.00	0.98–1.02
Sex (ref. female)	**1.77**	**1.12–2.81**	**2.00**	**1.18–3.36**
BMI: overweight/obese (ref. under-normalweight)	0.88	0.58–1.34	0.79	0.51–1.22
Current/past alcohol use (ref. never)	0.99	0.63–1.55	0.76	0.47–1.24
ALT (increasing IU/L)	1.00	0.99–1.00	1.00	0.99–1.01
AST (increasing IU/L)	1.00	0.99–1.00	0.99	0.98–1.00
Platelets (ref. > 100,000/μL)	**2.01**	**1.31–3.08**	**1.75**	**1.08–2.85**
Albumin (decreasing g/dL)	1.57	0.99–2.43	1.35	0.82–2.23
Bilirubin (increasing mg/dL)	0.98	0.87–1.12	0.84	0.60–1.18
INR (increasing unit)	**2.15**	**1.45–3.19**	**2.41**	**1.51–3.84**
HCV-genotype (3 vs others)	1.51	0.80–2.84	1.54	0.75–3.17
Diabetes	1.14	0.69–1.89	0.93	0.55–1.57
Anti-HBc+	1.02	0.63–1.65	1.05	0.63–1.76
Previous Interferon treatment	0.82	0.52–1.29	0.77	0.48–1.23
Esophageal varices	**1.85**	**1.20–2.85**	1.47	0.89–2.42
HCC	**2.32**	**1.20–4.49**	1.88	0.87–4.08
Previous decompensating event	**1.97**	**1.17–3.31**	1.12	0.60–2.11

## Discussion

This is one of a few multicentric cohort studies, representative of patients with chronic HCV infection in care in Italy that prospectively evaluated the medium-term outcomes following HCV eradication by DAA in consecutively enrolled patients with severe liver disease, based on HIV status. Regarding the HIV coinfection in our study population, HIV coinfected patients were at least 10 years younger than HCV monoinfected patients. This age difference potentially reflect the epidemiology of HIV infection mainly related with a later epidemic wave compared with the post transfusion and nosocomial epidemic wave of HCV monoinfection in Italy [21]. We found younger age, a higher prevalence of genotype 3, past or current alcohol abuse and HBV coinfection in HIV coinfected compared with HCV monoinfected patients. All these factors are known cofactors for liver disease progression [22–24] and could explain the more advanced liver cirrhosis in coinfected compared with monoinfected patients. However, after adjusting for all the above mentioned factors, only HIV coinfection was found independently associated with more severe liver damage before treatment. Based on this finding, our study could confirm that despite the younger age, coinfected patients have significantly more advanced liver cirrhosis compared with monoinfected patients. On the contrary, the possible longer time of infection and longer duration of cirrhosis, considering as surrogate marker of this the older age of monoinfected patients, could explain the higher HCC prevalence in monoinfected compared with coinfected patients prior to therapy [22].

Regarding the effectiveness of the DAA therapy we evaluated the liver disease related outcomes following the successful therapy according to HIV status. Previous studies have shown similar rate of recovery in liver function parameters in coinfected and monoinfected patients who achieved SVR after interferon-free, DAA-based treatment [25–27]. The peculiarity of our data is the outcome evaluation in patients with liver cirrhosis including also patients with C-P class B and C prior to viral eradication. Data in this group of patients are limited because patients with significant advanced liver disease were not specifically evaluated initially in clinical trials on DAA efficacy and were either excluded or, if included, their numbers were extremely low in real-life studies. Moreover, there are no long-term studies that prove the extent of clinical benefit for these patients.

In the present study, the successful DAA therapy for both HCV monoinfected and HIV/HCV coinfected patients was associated with improvement in C-P class in 85% of coinfected and in 64.6% of monoinfected patients, suggesting that viral eradication helps liver function recovery in the majority of patients with liver cirrhosis. Our results confirmed previously reported data in patients with liver cirrhosis after HCV eradication [28–31]. In particular, the percentage of C-P class improvement, observed in monoinfected patients, was similar to previous reports which evaluated patients with decompensated cirrhosis [32]. Other data from literature have reported a down-staging from C-P class B to A ranging from 31.6% [28] to 61.8% [32, 33] after a successful DAA treatment, suggesting that the variability in the methods used to select patients with cirrhosis in different published studies and the heterogeneity of patients included may influence the rate of improvement.

As far as our knowledge there are no sufficient data in the literature that evaluate the deterioration of liver function in terms of C-P score increase following the DAA therapy in coinfected vs monoinfected patients. In our study, although most patients with liver cirrhosis experienced improvement of liver function test following viral eradication, part of those kept the risk of liver disease progression regardless of viral eradication. We found that C-P class worsened in 8.2% HCV-monoinfected patients and in 5.4% HIV/HCV-coinfected patients. Male sex, increased INR and platelets counts lower than 100,000/μl, which are surrogate markers of severe liver damage and portal hypertension, were baseline predictors independently associated with C-P class worsening, while HIV coinfection was not an independent factor of liver disease worsening. In previous studies, additional analyses have suggested that patients above 65 years of age with reduced hepatic synthetic function were less likely to benefit from DAA therapy, however these factors were not sufficiently discriminative to identify a subgroup in which antiviral therapy should be deferred in favour of liver transplant [5].

Regarding the appearance of a decompensating event following viral eradication, we found that 11 coinfected patients (10.2%) and 114 monoinfected patients (9.2%) had a new decompensating event. These results were slightly higher compared with previous data that reported the appearance of a new decompensating event in 7.5% of patients [34]. Different liver disease severity prior to viral eradication could explain these differences [1, 6, 34, 35]. A total of 46.6% of coinfected and 45.8% of monoinfected patients with a history of hepatic decompensation before treatment start, maintained the risk of cirrhosis progression to decompensation after viral eradication. Our data were confirmed by another study that found about 36% of patients with severe portal hypertension remaining at high risk for decompensation [36]. Different risks in patients with different baseline characteristics have been indicated [1, 6], however there are lack of data in the literature regarding the rate of a first decompensating event following viral eradication. In this study a first decompensating event, in patients without a previous liver decompensation, was observed in 4.3% coinfected and in 4.8% monoinfected patients. A recent study reported a lower incidence of liver decompensation (0.9%), but the small sample size could justify the difference with our data [37].

Regarding the HCC occurrence, a similar cumulative HCC incidence in coinfected (2.2%) and monoinfected (3.9%) (*p*=0.38) patients after viral eradication, was reported in our recent study, suggesting that HIV coinfection is not associated with a higher probability of developing liver complications in successfully DAA treated patients with compensated cirrhosis [3].

Our findings confirm the existence of a point of no return, after which antiviral treatment may be too late to influence the natural history of HCV liver related disease, however more data are needed to better define this patient’s population.

## Conclusion

Viral eradication after DAA therapy represents a positive prognostic factor of liver function improvement, in particular in terms of C-P class. However, during a medium time of more than 2 years following the SVR achieved after the DAA treatment, this benefit was not extended in almost 10% of patients in whom liver disease progression continues regardless of viral eradication in both HCV monoinfected and HCV/HIV coinfected patients.

## Supplementary Information

**Additional file 1**

## Data Availability

The datasets used and/or analysed during the current study are available from the corresponding author on reasonable request.

## Abbreviations

BMI: Body mass index
CI: Confidence interval
C-P class: Child-Pugh class
DAA: Direct-acting antivirals
HCC: Hepatocellular carcinoma
HCV: Hepatitis C virus
HIV: Human Immunodeficiency Virus
HR: Hazard ratio
IFN: Interferon
INR: International normalized ratio
IQR: Interquartile range
OR: Odds ratios
PITER: Italian Platform for the study of viral hepatitis therapy
SVR: Sustained virologic response
SVR12: Sustained virologic response 12 weeks after treatment cessation
