# Supplementary material for: Liver function following hepatitis C virus eradication by direct acting antivirals in patients with liver cirrhosis: data from the PITER cohort
Source: BMC Infect Dis. 2021 May 4;21:413. doi: 10.1186/s12879-021-06053-3 (PMC8094561; doi:10.1186/s12879-021-06053-3)
Supplement: Supplementary file 1 — Additional file 1 [file 12879_2021_6053_MOESM1_ESM.pdf]

PITER Collaborating group:
Cesare MAZZARO, Manuela BERTOLA, Ornella SCHIOPPA (Clinical and Experimental Onco-Haematology Unit, CRO Aviano National Cancer Institute IRCCS, Aviano, Pordenone)
Antonio BENEDETTI, Laura SCHIADÀ, Monica CUCCO (Clinic of Gastroenterology and Hepatology, Marche Polytechnic University, Ancona)
Andrea GIACOMETTI, Laura BRESCINI, Sefora CASTELLETTI, Alessandro FIORENTINI (Institute of Infectious Diseases and Public Health, Marche Polytechnic University, Ancona)
Gioacchino ANGARANO, Michele MILELLA (Clinic of Infectious Diseases, University Hospital, Bari)
Alfredo DI LEO, Maria RENDINA, Fulvio Salvatore D'ABRAMO, Chiara LILLO, Andrea IANNONE, Mariano PIAZZOLLA (Gastroenterology Unit, University Hospital of Bari, Bari)
Gabriella VERUCCHI, Lorenzo BADIA (Clinic of Infectious Diseases and Microbiology Unit, Alma Mater Studiorum Bologna University, Bologna)
Fabio PISCAGLIA, Francesca BENEVENTO, Ilaria SERIO (Unit of Internal Medicine, Alma Mater Studiorum, University of Bologna, Bologna)
Francesco CASTELLI, Serena ZALTRON, Angiola SPINETTI, Silvia ODOLINI (Unit of Infectious and Tropical Diseases, University of Brescia Brescia)
Raffaele BRUNO, Mario MONDELLI (Infectious and Tropical Disease Unit, Fondazione IRCCS Policlinico San Matteo, Pavia)
Luchino CHESSA (Liver Unit, University of Cagliari. Cagliari)
Carlo TORTI, Chiara COSTA, Vincenzo PISANI, Vincenzo SCAGLIONE, Enrico Maria TRECARICHI (Unit of Infectious and Tropical Diseases, University "Magna Graecia", Catanzaro)
Anna Linda ZIGNEGO, Monica MONTI, Francesco MADIA (Department of Experimental and Clinical Medicine, Interdepartmental Centre MASVE, University of Florence, Florence)
Pier Luigi BLANC, Letizia ATTALA, Piera PIEROTTI, Elena SALOMONI, Elisa MARIABELLI (Infectious Disease Unit, S.M. Annunziata Hospital, Florence)
Teresa Antonia SANTANTONIO (Infectious Diseases Unit, Ospedali Riuniti, Foggia)
Ester Marina CELA (Department of Gastroenterology and Endoscopy, Ospedali Riuniti, Foggia)
Matteo BASSETTI, Giovanni MAZZARELLO, Anna Ida ALESSANDRINI, Antonio DI BIAGIO, Laura Ambra NICOLINI (Infectious Diseases Division, San Martino Hospital, Genoa)
Giovanni RAIMONDO, Roberto FILOMIA (Department of Internal Medicine, University Hospital of Messina, Messina)
Alessio AGHEMO, Rossella MELI (Internal Medicine and Hepatology Division, Humanitas Clinical and Research Center - IRCCS, Rozzano, Milan, Italy; Humanitas University, Department of Biomedical Sciences, Milan)
Adriano LAZZARIN, Giulia MORSICA, Stefania SALPIETRO (Department of Infectious Diseases, San Raffaele Hospital, Milan)
Massimo Galli (Department of Biomedical and Clinical Sciences 'Luigi Sacco', University of Milan, Milan)
Anna L. FRACANZANI, Erika FATTA, Rosa LOMBARDI (General Medicine and Metabolic Diseases, Fondazione IRCCS Ca' Granda Ospedale Maggiore Policlinico, Milan)
Pietro LAMPERTICO, Marta BORGHI, Roberta D'AMBROSIO, Elisabetta DEGASPERI (Division of Gastroenterology and Hepatology, Fondazione IRCCS Cà Granda Ospedale Maggiore Policlinico, Università degli Studi di Milano, Milan)
Massimo PUOTI, Chiara BAIGUERA, Federico D'AMICO (Infectious Diseases Unit, ASST Grande Ospedale Metropolitano Niguarda, Milan)
Maria VINCI (Gastroenterology and Hepatology Unit, ASST Grande Ospedale Metropolitano Niguarda, Milan)
Maria Grazia RUMI (Hepatology Unit, San Giuseppe Hospital, Milan)
Massimo ZUIN, Paola ZERMIANI (Liver and Gastrenterology Unit, ASST Santi Paolo e Carlo, Milan)

Pietro ANDREONE, Paolo CARACENI, Valeria GUARNERI (Department of Internal Medicine, University Hospital of Modena, Modena)
Erica VILLA, Veronica BERNABUCCI, Laura BRISTOT, Maria Luisa PARADISO (Department of Internal Medicine, Gastroenterology Unit, University of Modena and Reggio Emilia, Modena)
Guglielmo MIGLIORINO, Ilaria BERETTA, Alessandra GAMBARO, Giuseppe LAPADULA, Anna SPOLTI, Alessandro SORIA (Infectious Diseases, San Gerardo Hospital - ASST Monza, Monza)
Pietro INVERNIZZI, Antonio CIACCIO, Martina LUCÀ, Federica MALINVERNO, Laura RATTI (Gastroenterology and Hepatology, San Gerardo Hospital - ASST Monza, Monza)
Daniela Caterina AMORUSO, Federica PISANO, Ferdinando SCARANO, Laura STAIANO (Department of Hepatology, Gragnano Hospital, Naples)
Filomena MORISCO (Gastroenterology Unit, University of Naples Federico II, Naples)
Ivan GENTILE, Antonio Riccardo BUONOMO, Maria FOGGIA, Emanuela ZAPPULO (Department of Clinical Medicine and Surgery, University of Naples Federico II, Naples)
Alessandro FEDERICO (Department of Precision Medicine, University of Campania Luigi Vanvitelli, Naples)
Nicola COPPOLA, Caterina SAGNELLI, Salvatore MARTINI, Caterina MONARI (Infectious Diseases Division, University of Campania Luigi Vanvitelli, Naples)
Gerardo NARDONE, Costantino SGAMATO (Department of Gastroenterology, Federico II University of Naples, Naples)
Liliana CHEMELLO, Daniela STERRANTINO (Department of Medicine, University of Padua, Padua)
Francesco Paolo RUSSO, Alberto ZANETTO, Paola ZANAGA (Gastroenterology Unit, Department of Surgery, Oncology and Gastroenterology, University of Padua, Padua)
Francesco BARBARO (Infectious and Tropical Diseases Unit, Azienda Ospedaliera di Padova, Padua)
Antonio Craxì, Salvatore PETTA, Vincenza CALVARUSO, Luciano CRAPANZANO (Gastroenterolgy and Liver Unit, DiBiMIS, University of Palermo, Palermo)
Salvatore MADONIA, Erica Maria BRUNO (Department of Internal Medicine, Villa Sofia-Cervello Hospital, Palermo)
Anna LICATA, Simona AMODEO, Adele Rosaria CAPITANO (Internal Medicine, AOUP Paolo Giaccone, Palermo)
Carlo FERRARI, Elisa NEGRI, Alessandra ORLANDINI, Marco PESCI (Laboratory of Viral Immunopathology, Unit of Infectious Diseases and Hepatology, Azienda Ospedaliero-Universitaria di Parma, Parma)
Roberto GULMINETTI, Layla PAGNUCCO (Institute of Infectious Diseases, University of Pavia, Pavia)
Giustino PARRUTI, Paola DI STEFANO (Infectious Diseases Unit, Spirito Santo General Hospital, Pescara)
Maurizia Rossana BRUNETTO, Barbara COCO (Hepatology and Liver Physiopathology Laboratory and Internal Medicine, Department of Clinical and Experimental Medicine, University Hospital of Pisa, Pisa)
Romina CORSINI, Elisa GARLASSI (Infectious Diseases, Azienda Unità Sanitaria Locale – IRCCS di Reggio Emilia)
Massimo ANDREONI, Elisabetta TETI, Carlotta CERVA (Clinical Infectious Diseases, University of Tor Vergata, Rome)
Giuseppe GRASSI (Department of Medical Sciences, University of Tor Vergata, Rome)
Antonio GASBARRINI, Maurizio POMPILI (Internal Medicine, Gastroenterology and Hepatic Diseases Unit, Catholic University of Rome, Rome)
Gloria TALIANI, Elisa BILIOTTI, Martina SPAZIANTE (Infectious and Tropical Diseases Unit, Umberto I Hospital, "Sapienza" University, Rome)
Marcello PERSICO, Mario MASARONE, Andrea AGLITTI, Gemma CALVANESE (Internal Medicine and Hepatology Unit, University of Salerno, Salerno)
Marco ANSELMO, Monica MARTURANO (Infectious Diseases Unit, San Paolo Hospital, Savona)

Giorgio Maria SARACCO (Gastroenterology Unit, University of Turin, Turin)
Donatella IELUZZI (Clinical Unit of Gastroenterology, University Hospital of Verona, Verona)
